# Giant Vertebrobasilar Aneurysm: The Rule of Decompressive Craniectomy Previous to Endovascular Treatment

**DOI:** 10.7759/cureus.30187

**Published:** 2022-10-11

**Authors:** Pedro Teles, Vasco Carvalho, Rui Vaz, Maria Luís Silva, António Vilarinho

**Affiliations:** 1 Neurosurgery, Centro Hospitalar Universitário de São João, Porto, PRT; 2 Neurosurgery, Centro Hospitalar Universitário do Algarve, Faro, PRT; 3 Neurorradiology, Centro Hospitalar Universitário de São João, Porto, PRT

**Keywords:** endovascular stent, giant intracranial aneurysm, endovascular treatment, craniectomy, basilar artery aneurysm

## Abstract

Giant vertebral-basilar aneurysms are rare and represent 1% of intracranial aneurysms. Natural history and treatment are associated with severe clinical manifestations, such as ischemia, mass effect, hydrocephalus, and subarachnoid hemorrhage, leading to high mortality and morbidity. In this case, a 51-year-old male with no relevant medical history presented to the emergency department with severe pulsatile right temporo-occipital headache, radiating to the territory of the maxillary branch of the trigeminal nerve. Investigation revealed a giant unruptured vertebrobasilar aneurysm partially thrombosed. As treatment strategy, a suboccipital craniectomy was initially performed, and a week later, as a second stage, the patient underwent a stent placement from the V3 segment of the vertebral artery to the distal segment of the basilar trunk. Very few cases of this entity have been reported, and the endovascular treatment of this type of aneurysm is complex, with a high risk of mortality or morbidity, caused by thrombosis or by the inflammatory response secondary to the treatment, with compression of the brainstem. Decompressive craniectomy prior to endovascular treatment may play an important role in preventing life-threatening complications.

## Introduction

Giant aneurysms of the vertebrobasilar junction are a rare condition and represent an exceptional challenge to treat either using surgical or endovascular techniques [1,2]. Giant posterior circulation aneurysms are associated with high mortality rates, reaching 100% at five years, caused by rupture, spontaneous thrombosis with increased risk of stroke, and progressive growth responsible for mass effect, hydrocephalus, and brainstem mass effect [3-5]. Brainstem mass effect and patient age are the most important surgical prognosis factors, alongside aneurysm size, and patient comorbidities. The endovascular treatment offers a treatment without the morbidity/mortality of open surgery and is dependent on aneurysm size, neck-to-dome ratio, and presence of intraluminal thrombus [4-8]. We present a case of a combined treatment consisting of a posterior fossa decompressive craniectomy followed by endovascular treatment with stent placement from the V3 segment of the vertebral artery to the distal segment of the basilar trunk.

## Case presentation

**Figure 1:**
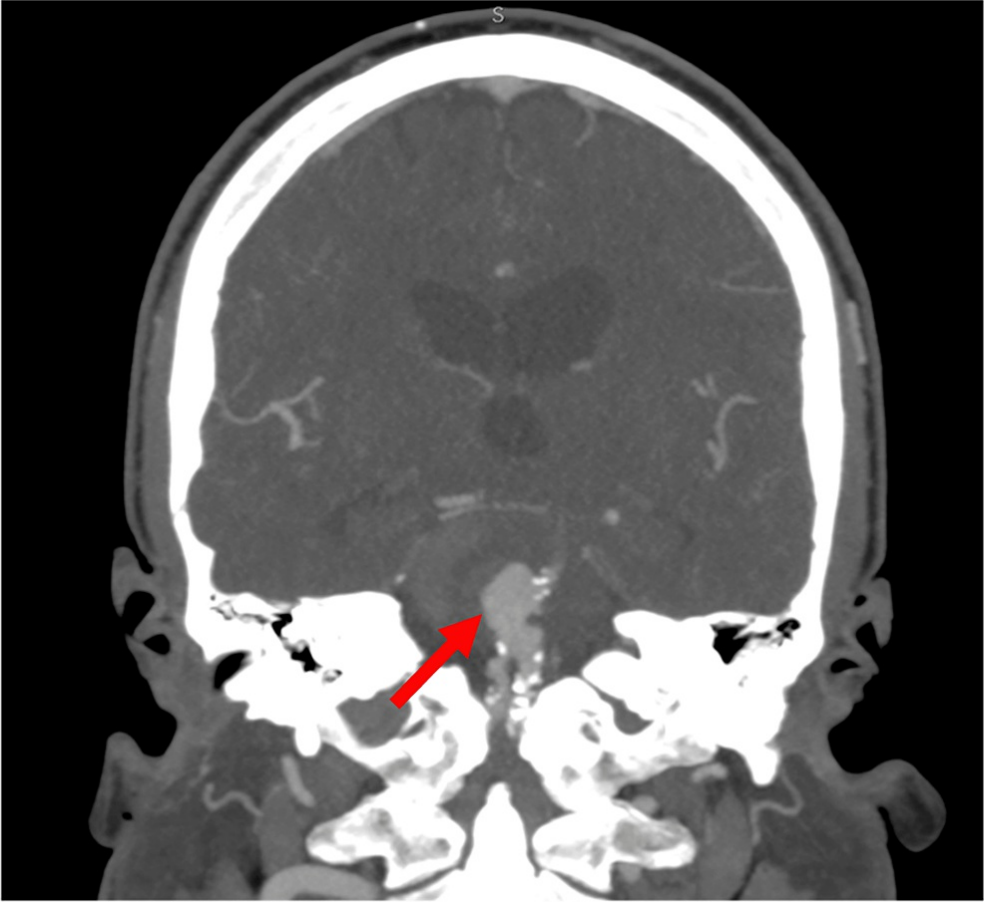
Computed tomography angiography revealing a large, partially thrombosed vertebrobasilar aneurysm (red arrow) with calcified atherosclerotic plaque occupying the distal two-thirds of the basilar artery, causing mass effect on the brainstem and fourth ventricle.

A 51-year-old male patient came to the emergency department due to severe pulsatile right temporo-occipital headache, radiating to the territory of the maxillary branch of the trigeminal nerve. Neurological examination on admission was normal, and the initial imaging investigation by computed tomography angiography revealed a large, partially thrombosed vertebrobasilar aneurysm with calcified atherosclerotic plaque occupying the distal two-thirds of the basilar artery, causing mass effect on the brainstem and fourth ventricle (Figure 1).

**Figure 2:**
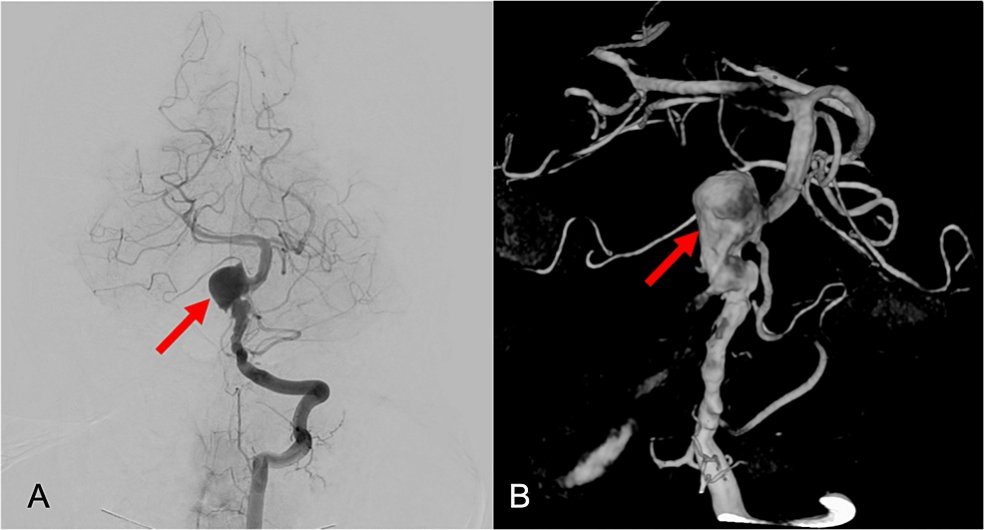
(A) Classic subtraction angiography and (B) reconstruction image revealing vascular dysplasia from the V4 segment of the vertebral artery to the middle segment of the basilar trunk with formation of a giant, partially thrombosed, aneurysmal sac on the right lateral aspect of the basilar trunk (red arrow).

Classic subtraction angiography revealed vascular dysplasia from the V4 segment of the vertebral artery to the middle segment of the basilar trunk with formation of a giant, partially thrombosed, aneurysmal sac on the right lateral aspect of the basilar trunk (Figure 2).

**Figure 3:**
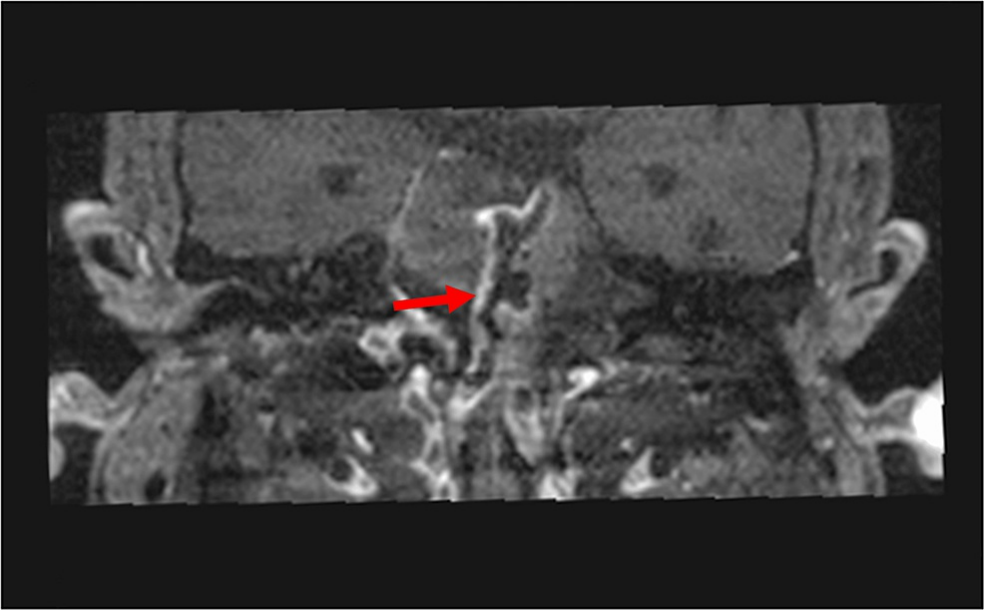
Magnetic resonance vessel wall image revealing linear and homogeneous peripheral enhancement (red arrow) in the vascular axis of the vertebrobasilar system and on the permeable component of the aneurysmal sac.

Further investigation with magnetic resonance vessel wall imaging revealed linear and homogeneous peripheral enhancement in the vascular axis of the vertebrobasilar system and on the permeable component of the aneurysmal sac (Figure 3).

During the hospital stay, the patient developed neurological worsening with mild right abducent nerve paresis and right side central facial paresis, remaining otherwise neurologically intact. After a multidisciplinary discussion and literature review, a combined treatment (decompressive craniectomy followed by endovascular treatment) was proposed.

Given the need for dual platelet antiaggregation therapy prior to endovascular treatment, surgery was planned to be performed in the first place and endovascular treatment performed five days after to exclude surgery-related bleeding and edema complications.

**Figure 4:**
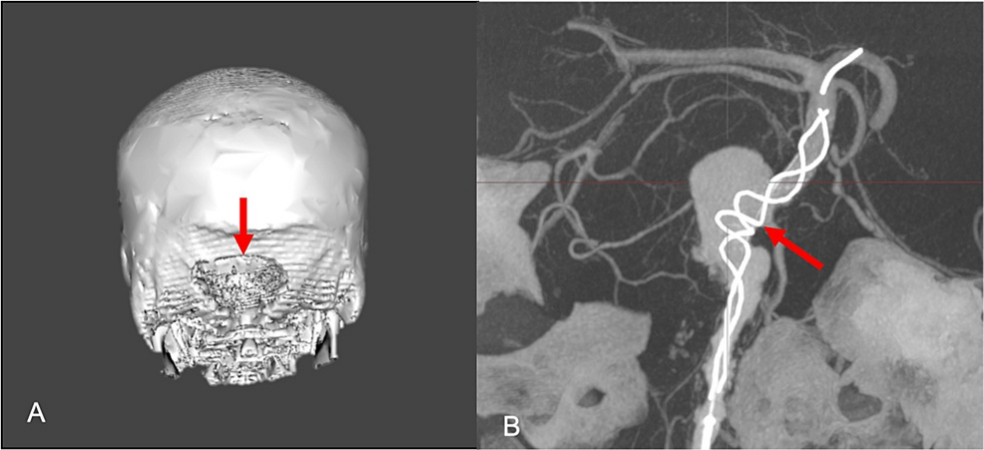
(A) Suboccipital decompressive craniectomy (red arrow). (B) Nitinol self-expanded stent placement from the distal segment of the basilar trunk to the V3 segment of the vertebral artery (red arrow).

In the first stage, a large suboccipital decompressive craniectomy was performed, followed by endovascular treatment with a self-expanded stent (LEO stent 4.5mm x 5mm) placement from the distal segment of the basilar trunk to the V3 segment of the vertebral artery, making use of the flow diversion effect and the possibility for stent-assisted coiling in the future. In this case, coiling was not attempted due to the exuberant vascular atheromatosis and the partially thrombosed aneurysmal sac (Figure 4).

The computer tomography angiography after treatment revealed adequate position of the endovascular stent, and a slight reduction in the aneurysm filling was observed compared to the pre-treatment study. There was no significant modification of the aneurysm size with similar mass effect exerted on the brainstem, and no images suggestive of acute ischemic vascular injury or intracranial hemorrhage were observed with only minimal herniation of the cerebellar parenchyma through the craniotomy bone defect being reported.

**Figure 5:**
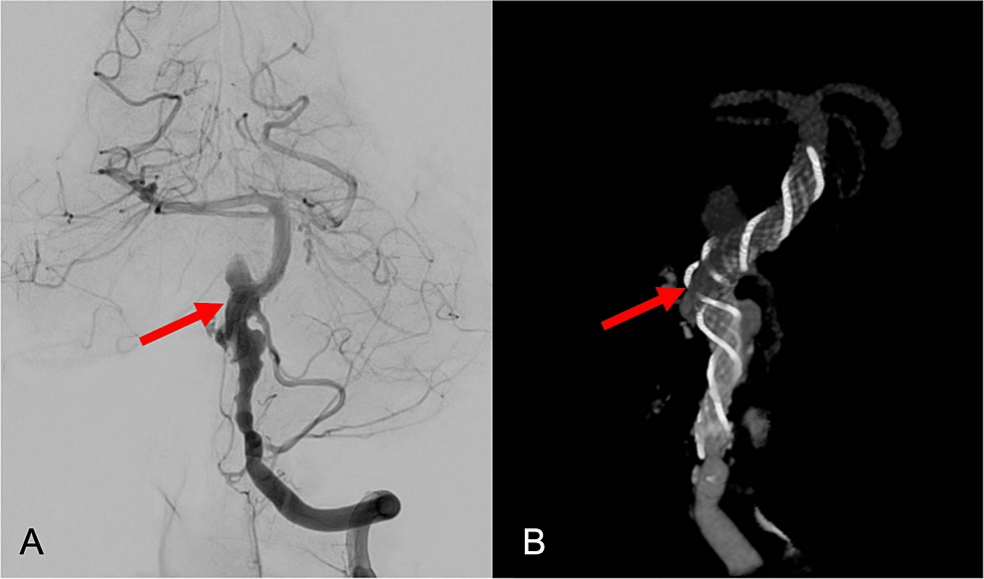
(A) Subtraction digital angiography revealing reduction in the dimensions of the residual aneurysm (red arrow). (B) Reconstruction image revealing adequate stent placement and patent vascular permeability (red arrow).

The patient had right abducent nerve paresis and right-sided central facial paresis and was discharged from the hospital a week after the endovascular treatment; He was referred to an outpatient clinic for further rehabilitation. At a six-month follow-up appointment, the patient presented with complete recovery from previous VI and VII cranial nerve paresis. Subtraction digital angiography revealed 70% reduction in the dimensions of the residual aneurysm with adequate stent placement and patent vascular permeability (Figure 5).

## Discussion

Vertebrobasilar aneurysms are a rare condition associated with poor outcomes. It usually presents with symptoms related to brainstem and lower cranial nerve compression, bleeding, hydrocephalus, and occlusion of vertebrobasilar perforating arteries [2,4,9]. If left untreated, neurological deterioration occurs mainly due to ischemic events, and ruptured and progressive mass effect in 65 to 100% of the cases, with mortality and morbidity rates as high as 100% [4].

Brainstem compression and patient age are the most significant surgical prognosis factors; additionally, aneurysm size and neurological status also play an important role. The endovascular treatment morbidity/mortality is less dependent on patient age but dependent on size, neck-dome ratio, and presence of intraluminal thrombus [3,4,10].

Recognition of aneurysms at high risk of rupture is paramount. PHASES score has been used to predict the five-year risk of rupture based on aneurysm morphology and patient characteristics, although it has not been validated worldwide.

The treatment goal is to prevent enlargement of the mass effect and create the conditions under which the aneurysm sac will shrink. However, this is difficult to obtain by surgical or endovascular means due to the presence of perforating arteries, endoluminal clots, and proximity to the brainstem and lower cranial nerves [2,3,9,11].

Magnetic resonance vessel wall imaging emerged as a promising instrument to identify unstable unruptured intracranial aneurysms [12]. Inflammation has been linked to aneurysm formation since Virchow in 1847, alongside hemodynamic stress and endothelial dysfunction. Prolonged inflammatory changes lead to continuous wall remodeling leading to atherosclerotic changes and accumulation of low-density lipoprotein increasing rupture risk [13-15].

The interpretation of vessel wall imaging of unruptured aneurysms is challenging and must be carried out with caution. Wall enhancement may be associated with the presence of inflammatory changes, but also with active macrophages, neovascularization, and decreased elastin, together increasing the risk of rupture [14,16].

Therefore, we performed a magnetic resonance vessel wall imaging, which revealed linear and homogeneous peripheral enhancement in the vascular axis of the vertebrobasilar system and on the permeable component of the aneurysmal sac. Based on these findings, a combined treatment strategy was proposed, consisting of a large suboccipital decompressive craniectomy followed by endovascular treatment with stent placement.

In previous cases, a decompressive suboccipital craniectomy would only be performed if complication from endovascular treatment occurred. This would mean that in case of a complication the patient would not only face a rapid deterioration from brainstem mass effect but also face an operation under dual platelet antiaggregation therapy.

Endovascular treatment with flow-modifying devices may lead to gradual thrombosis, recurrent intramural hemorrhages, and inflammation of the aneurysm wall, which can exacerbate the existing mass effect, leading to posterior fossa ischemia, brainstem compression, and edema [1-3]. Taking this into consideration, we performed a decompressive craniectomy five days prior to endovascular treatment, allowing not only to perform surgery under the best hemodynamic conditions but also to reduce the risk of mortality/morbidity from endovascular treatment.

## Conclusions

Decompressive craniectomy prior to endovascular treatment may play an important role in the prevention of severe neurological complications either by compression of the brainstem caused by aneurysm sac thrombosis or by secondary inflammatory response.
